# Deep Eutectic Solvent for Facile Synthesis of Mn_3_O_4_@N-Doped Carbon for Aqueous Multivalent-Based Supercapacitors: New Concept for Increasing Capacitance and Operating Voltage

**DOI:** 10.3390/ma15238540

**Published:** 2022-11-30

**Authors:** Nikola Zdolšek, Ivana Perović, Snežana Brković, Gvozden Tasić, Miloš Milović, Milica Vujković

**Affiliations:** 1 Department of Physical Chemistry, “Vinča” Institute of Nuclear Sciences—National Institute of the Republic of Serbia, University of Belgrade, Mike Petrovića Alasa 12-14, 11351 Belgrade, Serbia; 2 Institute of Technical Sciences of SASA, Knez Mihailova 35, 11000 Belgrade, Serbia; 3 Faculty of Physical Chemistry, University of Belgrade, Studentski Trg 12-14, 11158 Belgrade, Serbia

**Keywords:** deep eutectic solvent, porous carbon, Mn_3_O_4_- and N-doped carbon, supercapacitors, multivalent ion electrolyte

## Abstract

The capacitance and operating voltage of supercapacitors as well as their energy density have been increased by development of different materials and electrolytes. In this paper, two strategies, for the first time, were used to improve energy density: Mn_3_O_4_- and N-dual doped carbon electrode and aqueous mixture of multivalent ions as electrolyte. Mn_3_O_4_- and N-dual doped carbon was prepared by a novel and cost-effective procedure using deep eutectic solvent. XRD, XPS, and FTIR confirmed presence of Mn_3_O_4_ and nitrogen, while SEM and EDS elemental mapping showed micrometer-sized nanosheets with uniform distribution of C, O, N, and Mn atoms. Charge storage behavior of carbon was tested in aqueous multivalent-based electrolytes and their mixture (Ca^2+^-Al^3+^). Regarding both specific capacitance and workable voltage, the Ca^2+^-Al^3+^ mixed electrolyte was found as the best optimal solution. The calcium addition to the Al-electrolyte allows the higher operating voltage than in the case of individual Al(NO_3_)_3_ electrolyte while the addition of Al^3+^ ion in the Ca(NO_3_)_2_ electrolyte improves the multivalent-ion charge storage ability of carbon. As a result, the specific energy density of two-electrode Mn_3_O_4_@N-doped carbon//Al(NO_3_)_2_+Ca(NO_3_)_2_//Mn_3_O_4_@N-doped carbon supercapacitor (34 Wh kg^−1^ at 0.1 A g^−1^) overpasses the reported values obtained for Mn-based carbon supercapacitors using conventional aqueous electrolytes.

## 1. Introduction

Electrochemical supercapacitors (ES) can be considered energy storage devices of the future. Their main drawback is the low energy density, which can be improved in two ways: through the development of the high capacitance electrode materials and/or development of new electrolytes with an extended operating voltage.

Porous carbon electrode, doped with different heteroatoms and even oxides, tends to be potential electrode material with high capacitance, and therefore improve the energy density of ES [1,2,3]. Various carbon precursors have been introduced in order to simplify the synthesis procedure of doped carbon materials. Among these precursors are numerous biomasses [4,5,6], metal–organic frameworks [7], organic cross-linked polymers [8], etc. One of the newest precursors for facile (one-step) synthesis of different heteroatom doped carbon materials is ionic liquids (ILs) [9,10,11]. However, large scale application of ionic liquids in the carbon materials synthesis is limited due to the high price of ILs. On the other hand, carbon materials doped with different metal-oxides were successfully prepared using metal–organic frameworks (MOFs) [11]. However, the high cost of MOFs, as well as the complicated and usually toxic synthesis procedure of MOFs with relatively low carbon yield after carbonization, are defined as main disadvantages of MOFs application in carbon materials synthesis. Furthermore, the use of biomass waste as cheap precursor in the syntheses of carbons leads to several problems. The introduction of impurities and control of heteroatom doping can not be precisely controlled. Syntheses of metal-oxides-doped carbon are usually multi-step, complicated processes and sometimes include several steps and several techniques prior to final high temperature carbonization (such as hydrothermal carbonization, coprecipitation techniques, sol–gel, etc.) [12]. Including more than one technique in synthesis of material consequently increases the cost of synthesis procedure and limits its application on large (industrial) scale. Therefore, the main problem is to develop the “greener”, easiest, and cheapest synthesis route/procedure for carbon material production. For these reasons, all research is focused on developing a novel carbon precursor.

Deep eutectic solvents (DES) are a new generation of organic green solvents, composed of hydrogen bond acceptors and donors [13]. DES found numerous applications in different fields of science (analytical chemistry, pharmacy, food industry, etc.) due to their low price and almost negligible toxicity. In addition to this, DES found application in the synthesis of different nanomaterials and also in the synthesis of carbon materials. DES are the new generation of carbon precursors which satisfy most green chemistry principles in carbon material synthesis [1,14]. The use of DES offers one-step synthesis route for preparation of different heteroatom-doped carbon materials. Since DES usually contain nitrogen atoms, it is relatively easy to prepare nitrogen-doped carbon materials using only one DES (without additional substances). Additionally, DES can be made from various combinations of different organic compounds with different functional groups, which gives a possibility to produce carbon materials doped with heteroatoms other than nitrogen [15]. Therefore, DES act not only as carbon precursors, but also as doping agents. Other important properties of DES made them more suitable in the synthesis of carbon materials, compared to ILs, MOFs or organic polymers. DES have high viscosity and ionic strength and they can act as a soft template to produce functional micro/nano-sized particles with special morphology and properties [16]. Moreover, they have low vapor pressure which allows reactions at high temperature and ambient pressure (no need for high-pressure reactors) [17]. DES are eco-friendly and cheap solvents, that make possible a successful synthesis of carbons without additional organic solvents. All these benefits make DES suitable for industrial large-scale synthesis of carbon materials.

On the other hand, in order to increase operating voltage, different electrolyte formulations have been used. These electrolytes are usually organic, since this type of electrolytes shows larger operating voltage (around 2.7 V), than aqueous electrolytes (1 V) [18,19]. Due to their high toxicity, flammability, and high cost, modern electrochemistry leans towards the development of new, safer, non-organic aqueous electrolytes. In general, aqueous electrolytes can provide the higher specific capacitance of supercapacitors than organic electrolyte due to higher ionic conductivity. However, the main weak point of the aqueous supercapacitors is the low operating voltage, which do not exceed 1.2 V for the typical alkaline (KOH) and acidic (H_2_SO_4_) electrolytes. The higher operating voltage, up to even 2 V, can be achieved if neutral Li and Na sulfate electrolytes are used, but at the expense of lower capacitance. Recently, the use of acidic aluminum aqueous electrolyte [20] was found to be an interesting concept for extending operating voltage of “acidic” supercapacitor. Here, the addition of Ca(NO_3_)_2_ to the Al-based electrolyte was found as an effective way to boost both operating potential and capacitance of carbon-based supercapacitors. Namely, novel Mn_3_O_4_- and N-dual doped carbon material was developed using DES-assisted synthesis route and examined in an aqueous electrolyte of multivalent salts (Ca^2+^ and Al^3+^).

It is well known that different oxides are suitable electrode materials for supercapacitors, but in the last years Mn_3_O_4_ attracted special attention. It was studied for supercapacitor application due to low cost and good environmental compatibility [21]. However, the use of pure Mn_3_O_4_ electrode shows poor electrical conductivity, capacitance fading, low specific capacitance, low rate capacitance, and low long-cycle life [21,22]. Hence, different Mn_3_O_4_/carbon material electrodes have been developed in order to overcome the above-mentioned problems. Usually, the synthesis of Mn_3_O_4_/carbon materials is complicated multi-step procedure. It involves several steps to obtained Mn_3_O_4_ and a number of different chemicals need to be involved in the synthesis procedure. For example, Pan et al. introduced a freeze-drying step before final high temperature treatment [23], while other authors used hydrothermal synthesis step followed by freeze-drying to obtain Mn_3_O_4_ [24]. Our proposed synthesis method for obtaining Mn_3_O_4_- and N-dual doped carbon uses only carbonization of DES consisting of choline chloride-urea-MnCl_2_. This is a much simpler procedure compared to the above-mentioned methods. Used chemicals satisfy green chemistry principles, thus contributing to the reduction of the synthesis costs. Furthermore, the introduction of a new concept of mixing multivalent cations (aqueous mixture of Ca^2+^ and Al^3+^) was found to improve the capacitance performance of Mn_3_O_4_- and N-dual doped carbon.

## 2. Materials and Methods

### 2.1. Synthesis of Carbon Precursor

Deep eutectic solvents (DES) were used as carbon precursor. DES were prepared from three components: choline chloride (Acros Organics, purity 99%), urea (Sigma Aldrich, purity ≥ 99.5%) and MnCl_2_ (Sigma Aldrich, purity ≥ 99%). All three components was mixing in molar ratio 1:1:0.25 (choline chloride:urea:MnCl_2_) and preparation of DES followed two steps. In the first step, component was mixed at magnetic stirrer at 80 °C for 6 h. During this first step transparent liquid was formed, but MnCl_2_ was not completely dissolved. In the second step, mixture was treated in ultrasound bath at 60 °C for 6 h in order to completely dissolved MnCl_2_. After the second step, clear yellowish liquid (DES) was formed.

### 2.2. Carbon Material Synthesis

Carbon material was synthesized using prepared DES. DES were carbonized in tube furnace in N_2_ atmosphere at 800 °C (heating rate 5 °C min^−1^). After reaching 800 °C, the precursor was maintained at this temperature for 2 h, and cooled down to a room temperature (under N_2_ flow). The obtained carbon material (denoted as Mn_3_O_4_@N-doped carbon) was washed with deionized water to remove impurities.

### 2.3. Characterization of Carbon Material

Material was characterized using X-ray powder diffraction measurement (XRD), X-ray photoelectron spectroscopy (XPS), Fourier transformed infrared spectroscopy (FTIR), and scanning electron microscopy (SEM) with energy dispersive spectroscopy (EDS).

X-ray powder diffraction measurement was performed by Philips PW 1050 instrument. The diffraction intensity was recorded in the 2θ range of 10–70° with a step size of 0.02° and a counting time of 3 s per step.

X-ray photoelectron spectroscopy (XPS) was performed by SPECS Systems, equipped with XP50M X-ray source for Focus 500 and PHOIBOS 100/150 analyzer and AlKα source (1486.74 eV) at a 12.5 kV and 32 mA at a pressure of 9 × 10^−9^ mbar.

Fourier transformed infrared (FTIR) spectrum of prepared carbon was recorded using Nicolet iS5 FTIR spectrometer (Thermo Fisher Scientific, Waltham, MA, USA). FTIR spectra were measured in the range of 4000–400 cm^−1^ using the KBr pellet technique.

The morphology of carbon material was investigated using field emission scanning electron microscopy (FESEM), FEI SCIOS 2 Dual Beam electron microscope (Thermo Fischer Scientific, Waltham, MA, USA), operated at 10 kV, equipped with an energy dispersive X-ray spectroscopy (EDS) system.

### 2.4. Electrode Preparation and Electrochemical Measurements

The working electrode for electrochemical measurements was prepared by mixing carbon material and 5% Nafion (Sigma Aldrich) (ratio 95:5). The mixture was suspended in ethanol and sonicated for 1 h. Part of the homogenized mixture was transferred onto a glassy carbon electrode (GCE) and dried in an oven at 80 °C for 1 h. All electrochemical measurements (including cyclic voltammetry (CV), electrochemical impedance spectroscopy (EIS), and glavanostatic charge/discharge (GCD)) were performed using Gamry 1000E potenciostat/galvanostat in three-electrodes electrochemical cell with Pt as counter electrode, saturated calomel electrode (SCE) as reference electrode and modified GCE as working electrode at room temperature. Supporting electrolytes Al(NO_3_)_3_ and Ca(NO_3_)_2_ were used, as well as mixture of these electrolytes which contains Al(NO_3_)_3_ and Ca(NO_3_)_2_. Specific capacitance (F g^−1^) of electrode was calculated using cathodic or anodic charges integrated from CV voltammograms, Equation (1) [9]:

(1) $$C=\frac{\int I\cdot V\cdot dV}{m\cdot v\cdot \Delta V}$$

where *I* is the measured current, *V* is the potential, m is the mass of electroactive material layer onto the GCE, *v* is the applied scan rate, and Δ*V* is the used potential window.

GCD was performed in two-electrode configuration using two stainless steel electrodes covered with layer of prepared materials, separated with filter paper soaked with electrolyte and fixed face-to-face. Specific capacitance of electrode material was calculated according to Equation (2) [25]:

(2) $$C=\frac{2\cdot I\cdot t}{m\cdot \Delta V}$$

where *I* is the constant charge/discharge current, *t* is discharge time, Δ*V* is the potential window, and *m* is the mass of active material on the electrode.

## 3. Results and Discussions

Herein, we proposed a novel method for the synthesis of manganese oxide and nitrogen dual doped carbon material using deep eutectic solvents as carbon precursor for the use in multivalent-based supercapacitors. Carbonization of low-cost DES consisting of choline chloride, urea, and MnCl_2_ gives a possibility to prepare Mn_3_O_4_- and N-doped carbon material.

### 3.1. Characterization of Carbon Material

**Figure 1:**
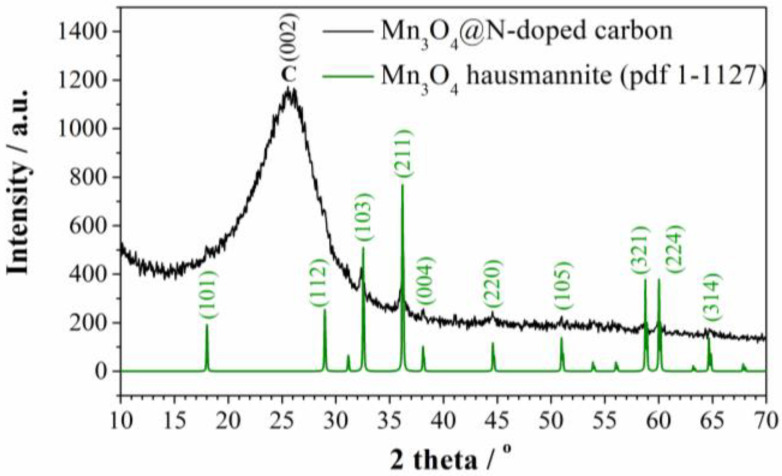
XRD diffractogram of Mn_3_O_4_@N-doped carbon.

The presence of manganese oxide was identified by XRD analysis. As can be seen from the diffractogram (Figure 1), there is a broad peak at 26° which corresponds to disordered graphitic carbon [26]. On the other hand, peaks corresponding to tetragonal Mn_3_O_4_ (Hausmannite, PDF No. 1-1127) indicate that MnCl_2_ was transformed into Mn_3_O_4_ phase during the carbonization process.

Furthermore, surface functional groups of Mn_3_O_4_@N-doped carbon were characterized by X-ray photoelectron spectroscopy (XPS). XPS showed presence of carbon, oxygen, nitrogen, and manganese on the carbon surface. Surface composition is presented in Table 1.

**Figure 2:**
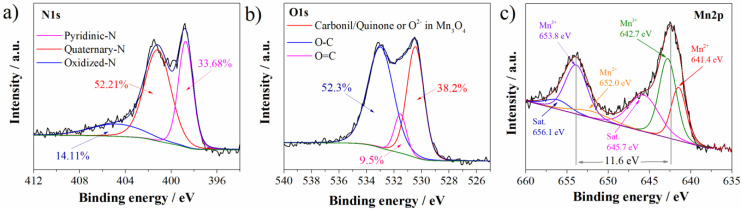
Deconvoluted (**a**) N1s, (**b**) O1s, and (**c**) Mn2p XPS spectra of Mn_3_O_4_@N-doped carbon.

Mn_3_O_4_@N-doped carbon sample is characterized by carbon matrix, with the presence of nitrogen, oxygen, and manganese. The presence of nitrogen and manganese is directly related to the presence of these groups in DES: nitrogen in choline chloride and urea and manganese in manganese chloride. Deconvolution of O1s, N1s, and Mn2p XPS spectra was performed in order to get insight into functional groups of these elements. Deconvoluted spectra are presented in Figure 2.

Regarding the nitrogen functionalities, three functional groups were detected in N1s spectrum (Figure 2a). Nitrogen is predominantly bonded as quaternary graphitic nitrogen observed at 401.2 eV (52.21% of total nitrogen content) [27,28]. Quaternary is followed by pyridinic nitrogen at 398.7 eV (33.68%) and oxidized nitrogen species at 404.8 eV (14.11% of total nitrogen content) [27,28]. As can be concluded, the highest percentage of total nitrogen is incorporated into graphitic microdomains.

Deconvoluted O1s spectrum (Figure 2b) showed the presence of three functional groups. Peak at 532.9 eV can be attributed to the C-O/C-OH functionalities (52.3% of total oxygen content) [29]. On the other side, Mn_3_O_4_@N-doped carbon sample showed a well-defined peak at 530.4 eV. This is characteristic binding energy of carbonil/quinone functional groups, but also this binding energy corresponds to O^2−^ in Mn_3_O_4_ [30]. These groups are present in lower content (38.18% of total oxygen content). In the lowest content of 9.5%, C=O/COOH functional groups at 531.5 eV are detected [30,31].

Mn2p spectrum (Figure 2c) showed Mn peaks corresponding to Mn2p_1/2_ and Mn2p_3/2_, respectively, with a splitting of 11.6 eV which is characteristic for Mn_3_O_4_ [29]. Furthermore, after deconvolution of Mn2p spectrum several characteristic Mn peaks appeared. Beside two satellite peaks at 656.1 eV and 645.7 eV, in the Mn2p spectrum, four peaks appeared: two peaks corresponding to the Mn^3+^ (653.8 eV and 642.7 eV) and two peaks to the Mn^2+^ (652.0 eV and 641.4 eV) [30].

**Figure 3:**
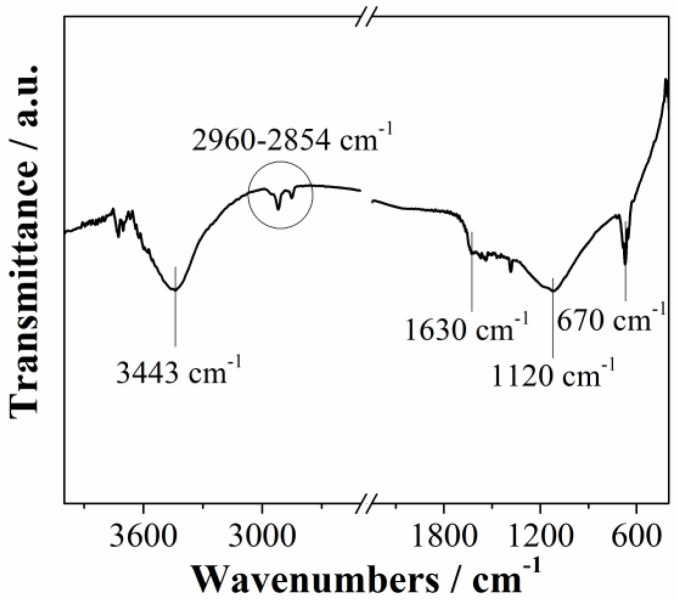
FTIR spectrum of Mn_3_O_4_@N-doped carbon.

Fourier transformed infrared spectroscopy (FTIR) of Mn_3_O_4_@N-doped carbon sample is presented in Figure 3. The spectrum showed typical bands of carbon-based materials. A broad band at around 3400 cm^−1^ corresponds to the OH stretching vibrations [32]. Bands between 2960 cm^−1^ and 2854 cm^−1^ indicate that the material has aliphatic structure [32]. The band at 1630 cm^−1^ is associated with C=O stretching vibration of carboxyl and carbonyl functional groups, while the band at 1120 cm^−1^ is associated with C–O stretching vibrations [33]. Peak at 670 cm^−1^ confirm presence of manganese oxide (Mn–O stretching modes) [34]. Obtained FTIR results are in agreement with XPS results.

**Figure 4:**
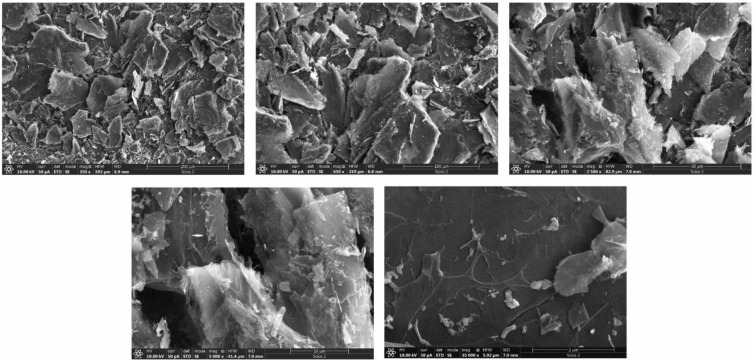
SEM micrographs of Mn_3_O_4_@N-doped carbon at five different magnifications (from 200 µm to 2 µm).

The morphology of Mn_3_O_4_@N-doped carbon was examined by scanning electron microscopy (SEM). Figure 4 shows SEM micrographs of Mn_3_O_4_@N-doped carbon at five different magnifications. As can be seen from these micrographs, the morphology of Mn_3_O_4_@N-doped carbon consists of micrometer-sized nanosheets/lumps with irregular particles.

**Figure 5:**
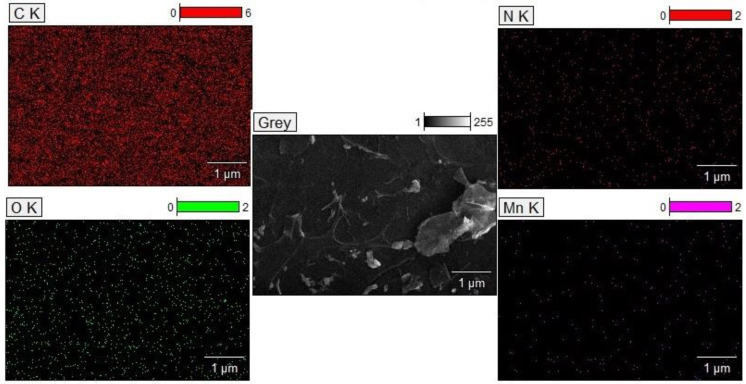
EDS elemental mapping analysis for Mn_3_O_4_@N-doped carbon: C mapping (**top left**), N mapping (**top right**), O mapping (**bottom left**), and Mn mapping (**bottom right**).

In addition to SEM, energy dispersive X-ray spectroscopy (EDS) confirmed the presence of C, N, O, and Mn, which is again in agreement with XPS results. Furthermore, EDS elemental mapping analysis of Mn_3_O_4_@N-doped carbon surface (Figure 5) shows uniform distribution of these elements (C, N, O, and Mn).

### 3.2. Electrochemical Investigation

Mn_3_O_4_@N-doped carbon was examined in multivalent-based aqueous electrolytes, Al(NO_3_)_3_ and Ca(NO_3_)_2_, as well as in their mixture consisting of Al(NO_3_)_3_ and Ca(NO_3_)_2_ (henceforth: Al^3+^-Ca^2+^ mixture).

**Figure 6:**
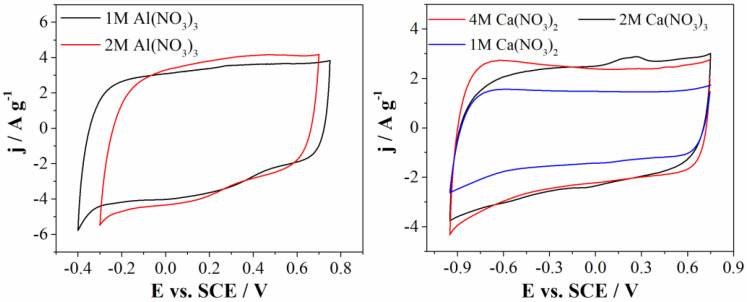
CVs of Mn_3_O_4_@N-doped carbon electrode in 1M and 2M Al(NO_3_)_3_ (**left**) and 1M, 2M, and 4M Ca(NO_3_)_2_ (**right**), recorded at 20 mV s^−1^.

Firstly, we investigated the effects of concentration of both electrolytes by means of cyclic voltammetry. Cyclic voltammograms of Al(NO_3_)_3_ and Ca(NO_3_)_2_ in different concentrations are represented at Figure 6.

Mn_3_O_4_@N-doped carbon electrode was examined in 1M and 2M (almost saturated solution) Al(NO_3_)_3_ electrolyte. As can be seen from Figure 6 (left), current densities in both electrolytes are the same, but hydrogen evolution in the case of 2M Al(NO_3_)_3_ started at more positive potential compared to 1M Al(NO_3_)_3_, thus shortening the potential window of Mn_3_O_4_@N-doped carbon electrode. Since the operating potential window is larger for 1M, it can be concluded that this is optimal concentration of Al(NO_3_)_3_. This difference in potential window could be consequence of a different pH values. pH value of 2M Al(NO_3_)_3_ is 0.78, while pH value of 1M Al(NO_3_)_3_ is 1.77. It is well known that hydrogen evolution reaction starts at more positive potentials at lower pH values. On the other hand, the more concentrated electrolyte may lead to the stronger solvation of ions and therefore more difficult water decomposition. However, the strong ion solvation occurs for both 1M and 2M concentrations of Al(NO_3_)_2_ (which are close to the saturated concentration), while the pH is the determining factor when starting the hydrogen evolution.

Furthermore, carbon electrode was examined in three electrolytes with different concentrations of Ca(NO_3_)_2_ (Figure 6 (left)). Mn_3_O_4_@N-doped carbon electrode showed the same curved shape and current densities in 2M and 4M Ca(NO_3_)_2_ electrolyte. Current densities in 1M Ca(NO_3_)_2_ are lower compared to current densities obtained in more concentrated solutions, probably due to low concentration of electrolyte ions. According to this, the optimal concentration for the calcium-based electrolyte is 2M Ca(NO_3_)_2_.

As can be seen from Figure 6, calcium nitrate showed less significant effects on the electrochemical window compared to Al(NO_3_)_3_. This behavior could be due to slight changes of the pH values of investigated calcium nitrate electrolytes. Furthermore, in neutral and near-neutral electrolyte, during cathodic polarization, HER occurs according to equation 2H_2_O + 2e^−^ → H_2_ + 2OH^−^. Namely, formed OH^-^ anion can attenuate the changes of pH at electrode/electrolyte interface and formation of H^+^ ions, resulting in local pH increase and consequently increasing the operating voltage [35].

**Figure 7:**
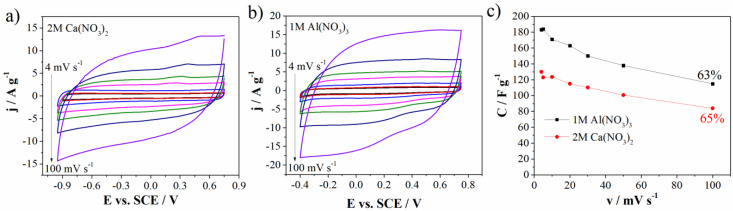
CVs of Mn_3_O_4_@N-doped carbon electrode in (**a**) 2M Ca(NO_3_)_2_, (**b**) 1M Al(NO_3_)_3_, and (**c**) specific capacitance values versus scan rate with capacitance retention.

Having in mind the above stated, further electrochemical experiments and detailed analysis were performed in 1M Al(NO_3_)_3_ and 2M Ca(NO_3_)_2_ electrolytes. Figure 7 shows cycling voltammograms of carbon electrode in two electrolytes 1M Al(NO_3_)_3_ and 2M Ca(NO_3_)_2_ at different scan rates ranging from 4 mV s^−1^ up to 100 mV s^−1^. One can see that calcium nitrate aqueous electrolyte (Figure 7b) provides the wider operating voltage window (1.7 V) than that of Al(NO_3_)_3_ (1.15 V) (Figure 7a). Again, this could be a consequence of the higher pH value of 2M Ca(NO_3_)_2_ (3.13) than that of 1M Al(NO_3_)_3_ (1.71).

As well as operating voltage window, Figure 7c shows specific capacitance values of Mn_3_O_4_@N-doped carbon electrode in two mentioned electrolytes at different scan rates and capacitance retention.

The inverse behavior of the specific capacitance was observed. In aluminum nitrate electrolyte, the carbon can deliver the capacitance value of 183 F g^−1^ and 163 F g^−1^ at 4 mV s^−1^ and 20 mV s^−1^, respectively, while corresponding capacitance values in 2M Ca(NO_3_)_2_ amount to 130 F g^−1^ at 4 mV s^−1^ and 115 F g^−1^ at 20 mV s^−1^ (Figure 7c). CV curves measured up to 100 mV s^−1^ almost retained the shape at high scan rates which indicates good transport of charges/ions within Mn_3_O_4_@N-doped carbon electrode [36]. Capacitance retention at 100 mV s^−1^ (in regard to 4 mV s^−1^) is also high and amounts to 65% for Ca(NO_3_)_2_ and 63% for Al(NO_3_)_3_.

All the stated evidence leads to a conclusion that different multivalent ions are responsible for different energy storage behavior of Mn_3_O_4_ carbon doped electrode. Al^3+^ ions hold the high values of the current and hence specific capacitance, while Ca^2+^ ions are responsible for wider potential window.

As mentioned before, the energy density of supercapacitors depends on operating window and specific capacitance value. In order to increase both and consequently increase the energy density, the novel approach based on electrolyte containing mixture of multivalent ions (Al^3+^ and Ca^2+^) was introduced. For this purpose in the next step, the energy storage on Mn_3_O_4_-doped carbon electrode was investigated in mixture of 1M Al(NO_3_)_3_ and 2M Ca(NO_3_)_2_ electrolyte (noted as Al^3+^–Ca^2+^ mixture).

**Figure 8:**
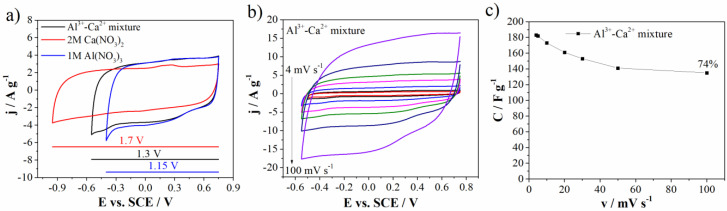
(**a**) CVs comparison of three electrolytes at 20 mV s^−1^, (**b**) CVs of Al^3+^-Ca^2+^ mixture at various scan rates, and (**c**) capacitance value with retention for Al^3+^-Ca^2+^ mixture.

Figure 8a shows comparative CVs of carbon electrode in individual (1M Al(NO_3_)_3_ and 2M Ca(NO_3_)_2_) and mixed electrolytes. One can see that the carbon current response, in the mixed electrolyte, is similar to the one in Al(NO_3_)_3_. It means that Al-ions dictate charge storage behavior of the carbon in the mixed electrolyte. This can be attributed to the faster transport of desolated Al^3+^ and H_3_O^+^ through the mesopores, due to their lower radius compared to desolvated Ca^2+^ ions [37]. This synergic effect of Al^3+^ and Ca^2+^ led to increase of both potential window up to 1.3 V in comparison to 1M Al(NO_3_)_3_ electrolyte and specific capacitance value when compared to the 2M Ca(NO_3_)_2_ electrolyte (183 F g^−1^ and 161 at 4 mV s^−1^ and 20 mV s^−1^, respectively). Furthermore, the capacitance retention obtained for mixture (73% at 100 mV s^−1^ relative to 4 mV s^−1^) was higher than the capacitance retention measured in individual electrolytes (Figure 8c).

According to our knowledge, the mixture of multivalent electrolyte ions in combination with Mn_3_O_4_-doped carbon electrode has never been investigated. For that reason, deeper insights into energy storage behavior needed to be attained for all investigated electrolytes. For this purpose, Trasatti analysis was used to differentiate and estimate the electrical double layer and pseudocapaictance contributions (EDLC and PC, respectively), according to the procedure described in references [38,39].

**Figure 9:**
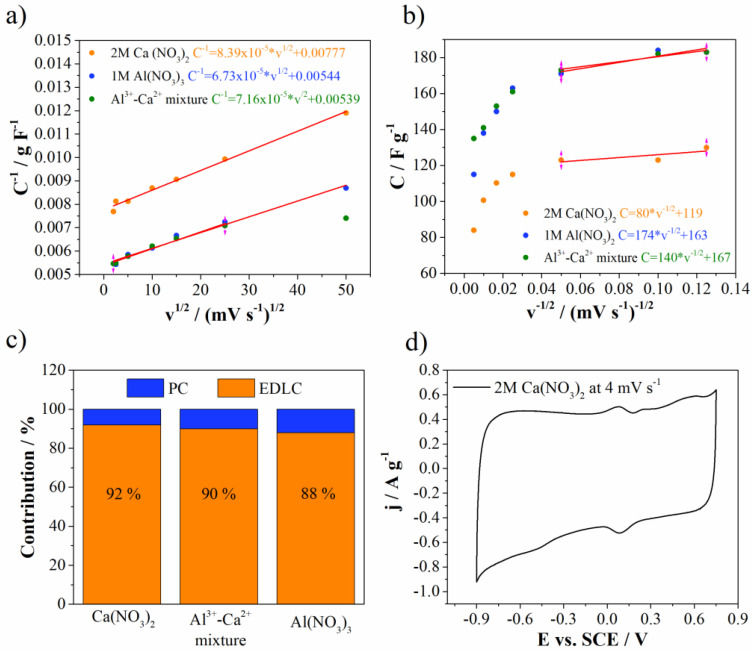
(**a**,**b**) Trasattis plots, (**c**) EDLC versus PC contribution and (**d**) CV curve of 2M Ca(NO_3_)_2_.

Figure 9 shows Trasatti plots together with EDLC and PC contributions for Mn_3_O_4_@N-doped carbon electrode in all investigated electrolytes. EDLC dominates carbon surface in all electrolytes. EDLC was higher for Ca(NO_3_)_2_ electrolyte (92%), while for Al(NO_3_)_3_ was the lowest (88%). The mixture of electrolytes showed EDLC contribution value of 90%, which perfectly lies between two mentioned values obtained for individual electrolytes. It is well known that carbon materials are very good electrical double layer materials for supercapacitors. On the other hand, Mn_3_O_4_ also contributes to EDLC formation due to adsorption and desorption of electrolyte cations on the surface of Mn_3_O_4_. Additional small redox peaks can be recognized in the CV of Mn_3_O_4_@N-doped carbon electrode in Ca(NO_3_)_2_ (Figure 4d) and belong to pseudocapacitive processes of Mn_3_O_4_. These redox peaks are more pronounced at slow scan rates (Figure 9d), while they are enwrapped by the capacitive current at higher scan rates (Figure 7a). These processes include the change of the Mn oxidation states over the surface upon anodic/cathodic sweep [40,41].

In addition to Mn_3_O_4_, the investigated electrode has oxygen functionalities which all contribute to pseudocapacitance reactions. Luo et al. proposed a possible redox mechanism of pseudocapacitance reaction between oxygen functional groups with Ca^2+^ ions, based on the large adsorption capacity of COOH groups for Ca^2+^ ions [42]. Namely, Ca^2+^ ions are prone to replace the protons from COOH during cathodic polarization, thus forming first -COOCa+ and then aldehyde group accompanied by Ca(OH)^+^ formation. The reverse, aldehyde → carboxylic conversion, occurs during anodic polarization. On the other hand, Al^3+^ ions interact indirectly with oxygen-containing groups (more precisely quinone groups) through pseudocapacitance reactions. During hydrolysis of Al salt, Al(H_2_O)_5_OH^−^ complex is formed and when this complex reaches C=O functional groups during cathodic polarization, OH^-^ ions are removed and charge transfer occurs [20]. The fact that Ca^2+^ and Al^3+^ ions mostly interact with different groups allows the utilization of their combined positive effects when they are mixed in the aqueous electrolyte.

**Figure 10:**
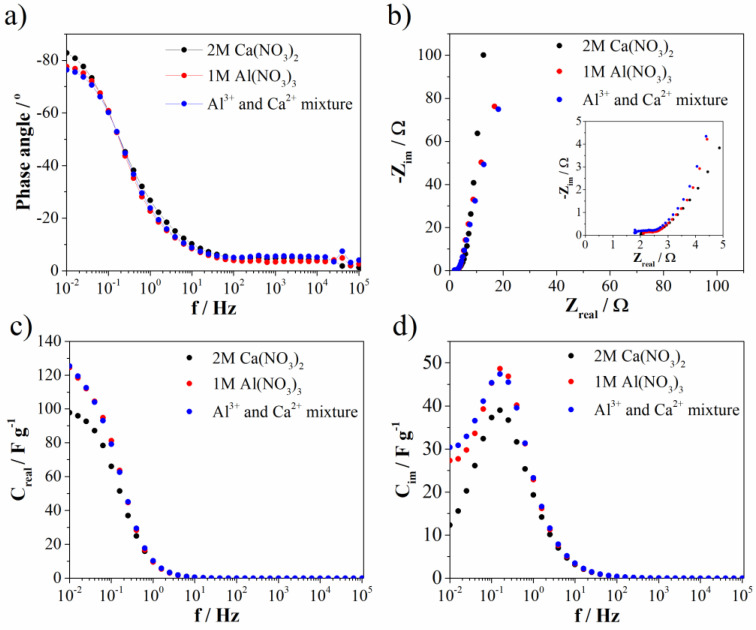
(**a**) Bode, (**b**) Nyquist plot, and (**c**,**d**) complex capacitance analysis.

Electrochemical impedance spectroscopy (EIS) (measured at 0.5 V vs. SCE) was used to investigate ion transport properties. Bode and Nyquist diagrams are represented at Figure 10a,b, while the complex capacitance analysis (Figure 10c,d) was done according to the reference [43]. As can be seen from the Bode diagram, the phase angle is highest for Ca(NO_3_)_2_ (and most close to −90°). Slightly lower, but the same value of phase angle was noted for Al(NO_3_)_3_ and Al^3+^-Ca^2+^ mixture when compared to the Ca(NO_3_)_2_ electrolyte. The phase angle close to −90° indicating ideal capacitance behavior, which is in good agreement with EDLC contribution. The EDLC contribution as well as phase angle are the highest for Ca(NO_3_)_2_ electrolyte. At the same frequency (low frequency region), the resistance from Nyqist diagram (Figure 10b) is highest for Ca(NO_3_)_2_, while the other two electrolytes showed again the same value. On the other side, ion charge transfer resistance (estimated according to the semicircle in high frequency region) increases in the order: Al(NO_3_)_3_ (0.47 Ω), Ca(NO_3_)_2_ (0.64 Ω), and Al^3+^-Ca^2+^ mixture (0.71 Ω). Bulk solution resistance of electrolytes increases in a different order: Al^3+^-Ca^2+^ mixture (1.79 Ω), Al(NO_3_)_3_ (2.1 Ω), and Ca(NO_3_)_2_ (2.0 Ω). All these small values of mentioned resistances imply high ionic conductivity at electrode/electrolyte interface and fast kinetics of Mn_3_O_4_@N-doped carbon electrode towards multivalent ions electrolytes. Furthermore, the trend of capacitance change (obtained by complex capacitance analysis) in the low frequency region (Figure 10c,d) followed the same trend found by CV for all investigated electrolytes. All electrolytes showed the maximum in C_im_ versus frequency plot. This maximum corresponds to the characteristic frequency (f_0_), i.e., the point where resistive and capacitive impedance are equal and the corresponding time constant (τ_0_ = 1/f_0_) defines border between resistive and capacitive behavior. Interestingly, Mn_3_O_4_@N-doped carbon electrode showed the same value for all investigated electrolytes (τ_0_ = 6.3 s). From all EIS measurements, it can be concluded that Mn_3_O_4_@N-doped carbon showed very low values of all mentioned resistances leading to potential application of this material in multivalent ions supercapacitors. Moreover, in the mixture of electrolytes, trivalent aluminum ion, despite lower concentration, has a dominant role in the term of ion transport properties compared to divalent calcium ion, since the mixture of electrolyte showed the same (or close) values of all parameters compared to Al(NO_3_)_3_.

**Figure 11:**
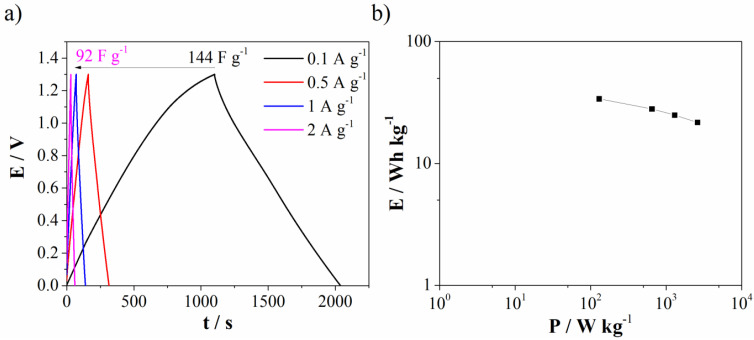
(**a**) GCD curves at different current density for Mn_3_O_4_@N-doped carbon in Al^3+^-Ca^2+^ mixture electrolyte and (**b**) Ragone plot.

Galvanostatic charge/discharge (GCD) of Mn_3_O_4_@N-doped carbon (two-electrode cell) was further used to test capacitance performances in real system with novel Al^3+^-Ca^2+^ mixture electrolyte. The specific discharge capacitance was found to be 144 F g^−1^ at 0.1 A g^−1^ and decrease of current density leads to decrease capacitance down to 92 F g^−1^ at 2 A g^−1^ (Figure 11a). Ragione plot (Figure 11b) shows energy density of 34 Wh kg^−1^ at low power density. The obtained energy density value is higher compared to values reported for similar Mn-carbon materials in standard most used electrolytes [44,45].

## 4. Conclusions

Deep eutectic solvent based on choline chloride, urea, and MnCl_2_ was used to synthesize carbon material. The use of the deep eutectic solvent provides easy and cheap route for synthesis of N- and Mn_3_O_4_-dual doped carbon. Carbon charactrization (XRD, XPS, FTIR, SEM with EDS elemental mapping) confirmed the presence of Mn_3_O_4_ and nitrogen. The obtained carbon was further tested in aqueous (inorganic) multivalent ions electrolytes using a novel strategy to improve charge storage behavior. Mn_3_O_4_-doped carbon electrode showed specific capacitance of 130 F g^−1^ at 4 mV s^−1^ and operating potential window of 1.7 V in aqueous Ca(NO_3_)_2_ electrolyte. On the other hand, investigated electrode showed lower operating window (1.15 V), but higher capacitance value of 183 F g^−1^ in aqueous Al(NO_3_)_3_ electrolyte. In order to increase the potential window, we designed novel approach based on mixed multivalent ions (Ca^2+^ and Al^3+^) electrolyte and Mn_3_O_4_-doped carbon electrode. In this novel approach, the potential window increased up to 1.3 V (compare to Al(NO_3_)_3_ electrolyte), while specific capacitance kept the value of Al^3+^ electrolyte. Investigation of charge storage mechanism using Trasatti analysis showed the electrical double layer formation is dominant. EDLC decreases in order: Ca(NO_3_)_2_ (92%), Al(NO_3_)_3_ (88%) and the mixture of electrolytes showed EDLC contribution (90%) perfectly between pure electrolytes. Furthermore, EIS showed that in a mixture of Ca^2+^ and Al^3+^, aluminum ions have a role since all the ion transport parameters showed the same or similar values as pure Al^3+^ electrolyte. All these synergic effects of mixture of multivalent ions (Ca^2+^ and Al^3+^) and Mn_3_O_4_- and N-doped carbon electrode (prepared from low-cost deep eutectic solvent) open up new views in electrochemical supercapacitor design.

## Figures and Tables

**Table 1:** Surface composition of Mn_3_O_4_@N-doped carbon determined by XPS.

	C	O	N	Mn
**Composition/at. %**	89.83	3.98	5.66	0.54

## Data Availability

Not applicable.
